# Overdose rescues by trained and untrained participants and change in opioid use among substance-using participants in overdose education and naloxone distribution programs: a retrospective cohort study

**DOI:** 10.1186/1471-2458-14-297

**Published:** 2014-04-01

**Authors:** Maya Doe-Simkins, Emily Quinn, Ziming Xuan, Amy Sorensen-Alawad, Holly Hackman, Al Ozonoff, Alexander Y Walley

**Affiliations:** 1 Clinical Addiction Research Education Unit, Section of General Internal Medicine, Boston University School of Medicine, 801 Massachusetts Avenue, Second Floor, Boston, MA 02118, USA; 2 Data Coordinating Center, Boston University School of Public Health, 801 Massachusetts Avenue, Third Floor, Boston, MA 02118, USA; 3 Department of Community Health Sciences, Boston University School of Public Health, 801 Massachusetts Avenue, Fourth Floor, Boston, MA 02118, USA; 4 Massachusetts Department of Public Health, 250 Washington Street, Boston, MA 02108, USA; 5 Boston Children’s Hospital, Center for Patient Safety and Quality Research, 300 Longwood Avenue, Boston, MA 02115, USA; 6 Harvard Medical School, Department of Pediatrics, 300 Longwood Avenue, Boston, MA 02115, USA

**Keywords:** Overdose, Opioids, Bystander naloxone, Rescue, People who use drugs

## Abstract

**Background:**

One approach to preventing opioid overdose, a leading cause of premature, preventable mortality, is to provide overdose education and naloxone distribution (OEND). Two outstanding issues for OEND implementation include 1) the dissemination of OEND training from trained to untrained community members; and 2) the concern that OEND provides active substance users with a false sense of security resulting in increased opioid use.

**Methods:**

To compare overdose rescue behaviors between trained and untrained rescuers among people reporting naloxone rescue kit use; and determine whether heroin use changed after OEND, we conducted a retrospective cohort study among substance users in the Massachusetts OEND program from 2006 to 2010. We used chi square and t-test statistics to compare the differences in overdose management characteristics among overdoses managed by trained versus untrained participants. We employed Wilcoxon signed rank test to compare median difference among two repeated measures of substance use among participants with drug use information collected more than once.

**Results:**

Among 4,926 substance-using participants, 295 trained and 78 untrained participants reported one or more rescues, resulting in 599 rescue reports. We found no statistically significant differences in help-seeking (p = 0.41), rescue breathing (p = 0.54), staying with the victim (p = 0.84) or in the success of naloxone administration (p = 0.69) by trained versus untrained rescuers. We identified 325 OEND participants who had drug use information collected more than once. We found no significant overall change in the number of days using heroin in past 30 days (decreased 38%, increased 35%, did not change 27%, p = 0.52).

**Conclusion:**

Among 4926 substance users who participated in OEND, 373(7.6%) reported administering naloxone during an overdose rescue. We found few differences in behavior between trained and untrained overdose rescuers. Prospective studies will be needed to determine the optimal level of training and whether naloxone rescue kits can meet an over-the-counter standard. With no clear evidence of increased heroin use, this concern should not impede expansion of OEND programs or policies that support them.

## Background

Drug overdose is a leading cause of premature, preventable mortality. In the United States, poisoning - 9 of 10 of which are drug overdoses - has surpassed motor vehicle crashes to become the leading cause of injury death [1,2]. In Massachusetts, USA, *opioid* related overdose exceeds motor vehicle crashes as the leading cause of injury death [3]. Drug overdose is also a major cause of mortality in Canada [4,5], Europe [6], Asia [7,8], and Australia [9].

One approach to opioid overdose prevention is to provide naloxone rescue kits to drug users. Naloxone is an opioid antagonist that has no abuse potential and reverses the effects of respiratory depression and decreased consciousness during an opioid overdose. Naloxone can be provided by prescription during the regular course of medical care [10-13], by pharmacist-initiated collaborative practice agreement [14], or by community-based overdose education with naloxone distribution (OEND) programs [15-26]. These programs target people who are at risk of opioid overdose and/or likely to be bystanders during an overdose to educate them on how to prevent an overdose from occurring, and to prevent opioid related over intoxication from progressing to a fatal overdose by seeking help, rescue breathing and administering naloxone. Between 1996 and 2010, over 50,000 potential bystanders were trained by OEND programs in the United States resulting in over 10,000 opioid overdoses reversed with naloxone [27]. The promise of this intervention has been recognized through endorsements by the United Nations Office on Drugs and Crime (UNODC) jointly with the World Health Organization (WHO) [28] US President’s Emergency Plan For AIDS Relief (PEPFAR) [29], the American Public Health Association (APHA) [30], state legislatures, public health departments and national programs [31]. We published an interrupted time-series analysis of the Massachusetts OEND program that demonstrated decreased opioid overdose death rates in communities that had implemented OEND compared to communities that had not, controlling for community level factors [32]. A simulation study of naloxone distribution to heroin users using conservative assumptions found an increase in Quality Adjusted Life Years (QALYs) and that naloxone distribution is cost-effective [33].

OEND programs have primarily been implemented among substance users who are at risk for overdose themselves and are likely to witness another person overdosing, and thus are in the position to help. While numerous studies have shown that trained laypersons are capable of recognizing and responding to opioid overdose events [16,19-24,26,32,34-36], the minimum length and content of these trainings is yet to be established. Trainings in these studies range from 5 minutes [32] to 8 hours [24]. Opinions about the right level of training range from the belief that naloxone rescue kits should be available over the counter without any mandated training [37], to the belief that an opioid overdose requires trained medical intervention and is not appropriate for layperson bystander response [38,39]. Once naloxone rescue kits are distributed into the community to people trained in overdose prevention, they are further disseminated through social networks to people who were not trained directly by the distribution programs (untrained). Whether these untrained potential bystanders successfully respond to overdoses with naloxone rescue kits is not known.

Clinicians, policy makers, and researchers have debated if providing people who use drugs with the skills to recognize and respond and the medicine (naloxone) to reverse opioid related overdoses, may increase opioid use or delay entry into addiction treatment by reducing interactions with emergency health care providers and the risk of adverse consequences of using drugs [38,40-43]. Although one prospective survey (N = 82) among people who inject drugs (PWID) found that 35% of respondents anticipated that he or she would feel comfortable using more heroin after receiving a naloxone rescue kit [44], no studies of existing OEND programs have demonstrated increased drug use by participants. One small study among PWID trained in OEND (N = 24) reported statistically significant decreases in heroin injection at 6-month follow-up [24], one (N = 47) reported that 53% of trained substance users decreased drug use at 3 months [26], and another among trained substance users (N = 22) reported no difference in drug use at follow-up [45].

Since 2006, the Massachusetts Department of Public Health’s Opioid Overdose Prevention Pilot program has provided OEND to potential overdose bystanders in numerous communities through local organizations providing varied types of services (e.g., HIV risk reduction services, outreach and case management, addiction treatment). Approximately two-thirds of the program participants have self-identified as a current or former drug user. In the current study, we sought to inform these two issues of whether “untrained” overdose bystanders can successfully respond to overdoses and how heroin use changes before and after training by using data reported by substance-using OEND program participants, including follow-up overdose rescue events and substance use data. The aims of this study were 1) to compare the management of overdose events by untrained rescuers to those by trained rescuers among people reporting naloxone rescue kit use; and 2) to assess how opioid use changed after receiving OEND at a large multisite OEND program in Massachusetts, United States over nearly five years.

## Methods

### Program description

In 2006, public health programs in Boston and Cambridge began providing overdose education and distributing intranasal naloxone rescue kits. In December of 2007, the Massachusetts Department of Public Health (MDPH), via a collaboration among public health and policy practitioners from the Commissioner’s Office, the Bureau of Substance Abuse Services and Office of HIV/AIDS, expanded OEND services to a total of eight agencies by 2010. The agencies provided regular individual and group OEND training sessions to potential opioid overdose bystanders via trained non-medical public health workers under a standing order from the program’s Medical Director. Settings include integrated public health prevention and screening programs for HIV, STIs, and viral hepatitis including needle exchange, low-threshold multi-service community service centers for injection drug users, detoxification programs, methadone maintenance treatment programs, other outpatient and residential addiction treatment programs, community meetings, emergency departments, homeless shelters, and home visits. The OEND agencies, the medical director, the MDPH, and subject area experts held monthly teleconferences on safety and quality assurance and met in person quarterly for quality improvement including data quality, interagency strategy transfer, and protocol and policy review. Periodic site visits by a master trainer occurred to ensure staff was sufficiently trained.

The MDPH OEND program uses the core components of the Skills and Knowledge on Overdose Prevention (SKOOP) training curriculum adapted for intranasal naloxone, originally developed by the Harm Reduction Coalition, and also incorporates elements of training developed by Chicago Recovery Alliance and the Drug Overdose Prevention and Education (DOPE) Project [20]. The curriculum [46] is delivered by trained nonmedical agency staff who must complete a four-hour course, a knowledge test and two trainings of potential bystanders supervised by a master trainer. The curriculum includes techniques in overdose prevention (i.e. minimizing polysubstance use, awareness of tolerance change) and management, such as how to assess for overdose, seek help, deliver rescue breathing, administer intranasal naloxone, post administration support, and specific techniques to avoid. Participants receive a naloxone rescue kit that includes instructions, two luer-lock, prefilled syringes with 2 mg/2 mL naloxone hydrochloride, and two mucosal atomization devices. Enrolled participants are instructed to deliver 1 mL (1 mg) to each nostril of the overdose victim. The second dose of naloxone in a rescue kit has dual roles. The first is that one dose may be insufficient for response if particularly strong opioids are involved in the overdose, such as unusually strong heroin or heroin adulterated with fentanyl. Secondly, because most opioid agonists have a longer half-life than naloxone, if overdose symptoms return, victims can be treated with the second dose. Depending on the enrollee’s previous experience and knowledge of overdose, the setting and the number of people being trained, trainings are provided in groups (no more than 10 people per staff member) or individually and can last between 5 and 60 minutes.

The program offers refills to participants on request. At the time of refill request, program staff collects data on the reason for the refill request and, if the program naloxone was used during an overdose rescue attempt, details about the overdose rescue.

### Study design and population

We conducted a retrospective cohort study using program data from the Massachusetts Opioid Overdose Prevention Pilot program among participants who reported any substance use in the 30 days prior to enrollment (N = 4,926). This program and sample have been described previously [32,47].

**Figure 1:**
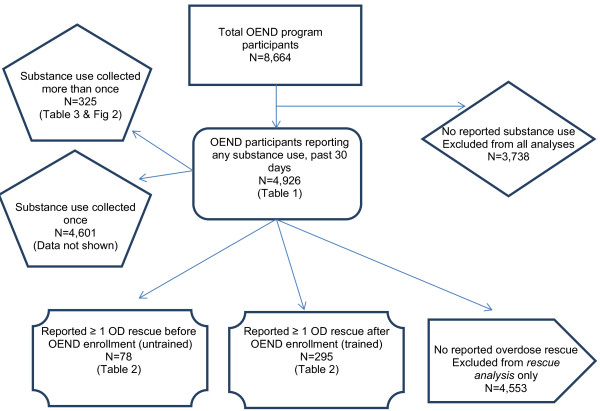
Flow diagram of study participants.

The training analysis (aim 1, N = 373, Figure 1) was conducted at the overdose rescue event level. We compared overdose rescues reported by people who were formally *trained* by OEND staff *prior* to reporting the rescue to those events reported by people who were trained by OEND staff at the time of rescue report, thus *untrained* by OEND staff at the time of overdose rescue. The *untrained* rescuers obtained the naloxone through social networks. Knowledge transfer about using naloxone at the time of obtaining naloxone from social network members ranges from none or minimal (for example: a rescuer who discovered an overdose victim and found the naloxone on the victim’s person) to extensive (for example: drug using partners who had discussed an overdose plan prior to an overdose event).

For the opioid use analysis (aim 2, N = 325, Figure 1), we restricted the study sample to program participants from whom we collected substance use information more than once between September 18, 2006 and December 31, 2010 because he or she was re-enrolled. These participants reported on their 30-day substance use at two or more time points, separated by at least 30 days.

### Data collection and measures

At enrollment, program staff created a unique program identifier for participants based on his or her birth date, the first three letters of his or her mother’s first name, first and third letter of his or her first and last names. Full names and addresses were not collected. This program identifier, in combination with age, gender and race, was used to eliminate duplicate enrollments and link enrollment and refill records. OEND staff also completed a questionnaire that included the participant’s demographics, lifetime overdose history, 30 day substance use history (number of days used out of the last 30 days), and 12 month detoxification program use. For substance use, route of administration was not asked. During a naloxone rescue kit refill, staff completed a questionnaire that included the reason for requesting a refill and, if it was because naloxone was used during an overdose rescue, questions about the overdose.

For aim 1, we used refill-rescue questionnaires administered by OEND staff when participants who presented to program sites to report an overdose rescue and request a naloxone rescue kit refill. (Figure 1) Staff was trained to define an overdose as an episode when an unresponsive victim had signs of respiratory depression after using substances. Other variables included in the refill-rescue questionnaire were used to describe overdose event characteristics; describe adherence to specific OEND training components, and; assess differences in the overdose events managed by the formally trained and untrained group. Overdose characteristic descriptive measures included: the relationship to the person who overdosed, the setting (public or private), the number of naloxone doses used, and whether naloxone was successful. Naloxone administration was considered successful if the victim’s unresponsiveness and respiratory depression improved and the person survived. Overdose response items on the questionnaire that we defined as adherent to OEND training were sternal rub (assess for overdose), 911 called (seek help), rescue breathing, administer naloxone, staying with victim (post naloxone support). Overdose response items on the questionnaire that we defined as non-adherent to OEND training techniques were slap, ice/water applied, and salt/cocaine injection.

We examined the dates of the enrollment and refill questionnaires to identify the subset of program participants who were not trained by OEND staff prior to an overdose rescue. (Figure 1) Participants who reported administering naloxone during an overdose rescue attempt, but who had not previously completed an enrollment form, were defined as untrained at the time of the rescue attempt. Those who reported administering naloxone during a rescue attempt who had been enrolled previously were defined as trained. Any subsequent rescue reports by people who were untrained at the time of first report were transferred to the trained category, as the standard practice at each site was to enroll and train participants at the time of the first rescue report, if they had not been previously enrolled.

For aim 2, we used pairs of enrollment questionnaires completed by participants who had been enrolled in OEND more than once separated by at least 30 days. (Figure 1) These “repeat enrollments” occurred because OEND services were delivered by eight agencies, none of which shared lists of enrollment codes. Thus, staff at one program did not know if the participant completed the enrollment questionnaire at another site, so when an individual presented for the first time at an agency for OEND services, an enrollment questionnaire was completed regardless of whether the individual had previously enrolled at another site. Additionally, when OEND services were provided during outreach, a staff person might not have access to the participant code list to determine if the program participant had been enrolled. Therefore, the staff re-enrolled participants whose enrollment status was uncertain, creating a subset of repeat enrollers from whom information was collected more than once. Among this group, we are able to report on substance use behavior in the time period preceding receiving OEND services the first time, as well as, a later time period after having received training and a naloxone rescue kit.

To examine frequency of use for each individual substance (heroin, benzodiazepine/barbiturate, cocaine, methadone, alcohol, buprenorphine, and other opioids), we created the following four categories from the enrollment questionnaire: those reporting no use in the past 30 days; 1–10 days of use out of the past 30; 11–20 days of use and; 21–30 days of use. We created a measure of number of substances used in previous 30-day period, defined as the sum of the number of substances (heroin, methadone, buprenorphine, other opioids, cocaine, alcohol, benzodiazepine/barbiturate and methamphetamine) that respondents reported consuming at least one day out of the past 30 days. We further categorized number of substances used as increased when a person reported a higher number at the second enrollment, compared to the first enrollment; decreased when a person reported a lower number at second enrollment compared to first enrollment, and; no change when the number of substances used stayed the same. We also measured the time between enrollments.

This study was approved by the Institutional Review Boards of Boston University and the Massachusetts Department of Public Health. This study uses de-identified existing program data and written informed consent was not obtained. Cells with a count of 5 or less have been suppressed as an identity protection measure.

### Analysis

Data from enrollment and refill questionnaires collected by the eight agencies were stored and maintained in a central MDPH OEND program database. From this database, we derived a de-identified dataset from which means, frequencies and proportions were calculated using SAS version 9.3 statistical software.

For the analyses for aim 1, we employed t-tests for continuous variables and chi square tests for categorical variables to assess the difference between participants who never rescued, those that rescued after enrollment (trained) and those that rescued before enrollment (untrained), as well as, to assess the difference between overdose rescue characteristics between those managed by trained versus untrained program participants.

For the analyses for aim 2, we used t-tests for continuous variables and chi square tests for categorical variables to assess the difference between repeat enrollers and one-time enrollers. To compare substance use among repeat enrollers at first and second enrollment, we employed the Wilcoxon signed rank test to compare the median difference of the number of days of each substance use and the number of substances used between two repeated measures of the repeat enrollers sample. As part of a sensitivity analysis to determine whether the time between enrollments was a predictor of drug use at second enrollment, we used linear regression analysis with days of use at second enrollment as the dependent variable and days of use at first enrolment and time between enrollments as independent variables.

## Results

### Characteristics of the study population

Between September 18, 2006 and December 31, 2010, 8,664 participants were trained to prevent, recognize and respond to an overdose, including receiving a naloxone rescue kit. We excluded 3,738 participants who did not report any substance use at any enrollment. Of the 4,926 participants who reported any substance use, 4,553 (92.4%) never reported an overdose rescue, 295 (6.0%) trained participants reported at least one rescue and 78 (1.6%) untrained participants reported at least one rescue with naloxone prior to being enrolled in the OEND (Table 1).

**Table 1:** Characteristics of overdose education and naloxone distribution program participants who reported any substance use, Massachusetts, 2006-2010

	**No rescue reported**	**Trained rescuers**	**Untrained rescuers**
	**N = 4553**	**N = 295**	**N = 78**
Age mean (std dev)	34.1 (10.8)	35.1 (10.6)	35.0 (10.3)
Female and MtF	35% (1579/4479)	34% (99/289)	42% (33/78)
Race/Ethnicity			
Hispanic	14% (643/4511)	11% (31/293)	17% (13/78)
White, Non-Hispanic	77% (3463/4511)	80% (235/293)	81% (63/78)
Black/African American, Non-Hispanic	5.5% (249/4511)	6.1% (18/293)	**
Other, Non-Hispanic	3.5% (156/4511)	3.1% (9/293)	**
Enrollment location type			
Inpatient detoxification	42% (1554/3730)	24% (35/144)	21% (13/61)
Needle exchange	17% (635/3730)	31% (45/144)	41% (25/61)
Drop-in center	13% (503/3730)	26% (37/144)	33% (20/61)
Other	28% (1038/3730)	19% (27/144)	**
Opioid use, previous 30 days			
Heroin only	48% (2199/4553)	60% (181/295)	72% (56/78)
Any other opioid	30% (1372/4553)	27% (81/295)	22% (15/78)
No opioid use	22% (982/4553)	13% (38/295)	**
Polysubstance use, previous 30 days	77% (3515/4553)	80% (235/295)	79% (62/78)
Attended detoxification, past year	63% (2769/4397)	53% (146/278)	55% (41/75)
Released from incarceration, past year	26% (1140/4389)	29% (80/275)	16% (12/75)
Any homelessness, past year	37% (1592/4291)	38% (102/272)	38% (28/74)
Ever had a nonfatal overdose	51% (2248/4425)	63% (178/281)	68% (51/75)
Ever witnessed an overdose	76% (3378/4464)	86% (249/288)	94% (72/77)
Reported >1 overdose rescue	--	34% (101/295)	24% (19/78)
mean (std dev)		2.9 (1.6)	2.6 (1.3)

Denominators less than the total number for each group are due to missing information.

**Cells with values less than 5 suppressed.

Prevalence of overdose risk factors including homelessness, polysubstance use, tolerance changes associated with supervised withdrawal procedures (detox) and incarceration, previous nonfatal overdose and lifetime witness of overdose were present in both participants who reported rescues and those who did not at similar rates. Participants who reported a rescue were less likely to have attended detox in the previous year (p < 0.001) and more likely to have had a nonfatal overdose (p < 0.001) and to have witnessed at least one overdose (p < 0.001). Participants who reported a rescue were more likely to report using only heroin than the participants who never reported a rescue (p < 0.001). Participants who did not report a rescue were more likely to be enrolled at a detox location, while those who did report a rescue were more like to have been enrolled at needle exchange programs or drop-in centers for PWID (p < 0.001).

Characteristics of one-time enrollers (n = 4,601) and repeat enrollers (the subset of 325 participants who had at least two enrollments that were separated by at least 30 days- Figure 1) were similar, though repeat enrollers were more likely than one-time enrollers to have attended detox in the past year (70% vs 62%, p < 0.001), have a personal history of overdose (63% vs 51%, p < 0.001), and report an overdose rescue using program naloxone (17% vs 7%, p < 0.001). (Data not shown).

### Overdose rescues by trained vs. untrained rescuers

Of the 373 substance-using OEND program participants who reported an overdose rescue (Figure 1), 34% of the trained and 24% of the untrained participants reported more than one overdose rescue with a mean of 2.9 and 2.6 rescues among those with more than one in each group respectively (Table 1) for a total of 599 overdose rescue events using OEND program naloxone. (Table 2) Most (67% & 69%, p = 0.92 for trained and untrained rescuers, respectively) of the overdose victims were friends of the rescuer. Overdoses most commonly occurred in a private setting (79% & 70%, p = 0.13 for trained and untrained rescuers, respectively) and were successfully managed with only one dose of naloxone (52% & 61%, p = 0.06 for trained and untrained rescuers, respectively). About half of the time the victim received rescue breathing (47% & 52%, p = 0.54 for trained and untrained rescuers, respectively) and about one quarter of the time, 911 was called or emergency medical services were present (23% & 27%, p = 0.41 for trained and untrained rescuers, respectively). Most of the rescuers stayed with the victim and/or turned care over to emergency medical professionals (89% & 89%, p = 0.84 for trained and untrained rescuers, respectively). The most common form of stimulation that the rescuer performed was a sternal rub – a recommended technique in OEND training– followed by slapping and applying ice or cold water, which were discussed during training and not recommended. None of the rescuers reported injecting salt water or cocaine.

**Table 2:** Overdose rescues reported by substance using bystanders in Massachusetts, 2006-2010

	**Rescues after training**	**Rescues before training**	p-value*
	**N = 508**	**N = 91**	
Relationship to overdose victim			0.92
Friend	67% (341/508)	69% (63/91)	
Partner/family	12% (62/508)	13% (12/91)	
Stranger	9.1% (46/508)	8.8% (8/91)	
Client/patient	**	**	
Self	10% (53/508)	8.8% (8/91)	
Declined	**	**	
Overdose setting			0.13
Private	79% (395/498)	70% (62/89)	
Public	20% (100/498)	29% (26/89)	
Declined	**	**	
Number of doses used			0.06
1	52% (244/468)	61% (52/85)	
2	43% (201/468)	39% (33/85)	
3+	4.9% (23/468)	0.0% (0/85)	
Naloxone successful	97% (295/303)	96% (54/56)	0.70
911 called or emergency personnel present	23% (119/508)	27% (25/91)	0.41
Rescue breathing performed	47% (166/350)	52% (34/66)	0.54
Stayed with victim until alert or help arrived	89% (445/498)	89% (78/88)	0.84
Sternal rub	63% (222/350)	62% (41/66)	0.84
Slap	38% (134/350)	35% (23/66)	0.60
Ice or water	9.4% (33/350)	14% (9/66)	0.30
Salt or cocaine shot	0.0% (0/350)	0.0% (0/66)	--

Denominators less than the total number for each group are due to missing information.

*Categorical variables are compared using a chi square test.

**Cells with values less than 5 suppressed.

We found no statistically significant differences in the overdose event characteristics or actions taken during the overdose where the rescuer was previously trained by OEND staff compared to the events where the rescuer was not previously trained.

### Changes in opioid use among participants enrolled more than once

The mean number of days between the first enrollment and the second enrollment was 364 (median 288), with a maximum of 1473 and a minimum of 30 days between the times of information collection.

**Figure 2:**
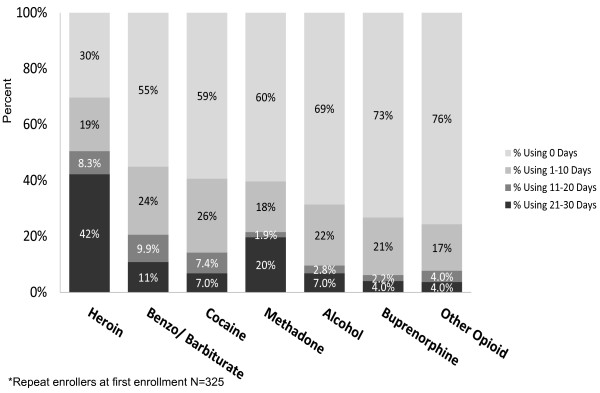
Percentage of overdose education and naloxone distribution program participants* reporting substance use, past 30 days.

Among participants with more than one enrollment, heroin was the substance used most commonly with 70% reporting any heroin use in the past 30 days, and 42% reporting heroin use in at least 21 of the last 30 days. (Figure 2) Less than half reported using benzodiazepines/ barbiturates (45%), cocaine (41%), methadone (40%), alcohol (31%), buprenorphine (27%), or other opioids (24%). Among people who reported taking methadone, at least 21 days of use was common, whereas among participants who reported any use of other substances, most participants used 1–10 days out of the last 30 days. These patterns of use were similar for those who were one-time enrollers and for the first and second enrollment among the multiple enrollers (data not shown).

Among the 325 participants from whom 30 day substance use data were collected twice, 38% had decreased days using heroin and 35% had increased days using heroin. (Table 3) More participants decreased the number of days of heroin (38% v 35%, p = 0.52), other opioids (19% v 18%, p = 0.51), cocaine (30% v 26%, p = 0.41), and alcohol (22% v 21%, p = 0.86) use than increased, though these findings were not statistically significant. More participants increased the days of methadone (26% v 22%, p = 0.72) and buprenorphine (22% v 20%, p = 0.31) than decreased, though this was not statistically significant. There was a statistically significant difference in participants who increased than decreased days using benzodiazepine/barbiturates (30% v 23%, p = 0.004). Forty per cent increased the number of substances used in the past 30 days, 38% decreased the number of substances, and 21% reported no change (p = 0.65). Time between enrollments was not a significant predictor of drug use at second enrollment (data not shown).

**Table 3:** Change in substance use among overdose education and naloxone distribution program participants between first and second enrollment- number of days and substances used, past 30 days

**N = 325**	**Increased**	**Decreased**	**No change**	p-value*
Heroin	115 (35%)	122 (38%)	88 (27%)	0.52
Methadone	84 (26%)	70 (22%)	171 (52%)	0.72
Buprenorphine	73 (22%)	66 (20%)	186 (58%)	0.31
Other opioids	59 (18%)	62 (19%)	205 (63%)	0.51
Cocaine	83 (26%)	96 (30%)	146 (44%)	0.41
Alcohol	69 (21%)	70 (22%)	186 (57%)	0.86
Benzo/Barbiturate	99 (30%)	74 (23%)	152 (47%)	0.004
Number of substances** used	131 (40%)	125 (38%)	69 (21%)	0.65

*Wilcoxon signed rank test which compares the median difference between two repeated measures among the repeat enrollers.

**Participants were asked about use of heroin, methadone, buprenorphine, other opioids, cocaine, alcohol, benzodiazepine/barbiturate and methamphetamine.

## Discussion

This study describes the implementation of a large public health department-sponsored overdose education and intranasal naloxone distribution program among 4,926 Massachusetts participants who reported substance use at enrollment. We found no significant evidence of differences in the management of overdose events by untrained rescuers compared to trained rescuers and no significant evidence of overall change in opioid use after receiving naloxone rescue kits.

### Trained versus untrained overdose rescuer events

We describe a previously unstudied subset of overdose rescue reports - those performed by people who had not been trained by OEND staff at the time of using naloxone during an overdose. Although we have categorized them as *untrained,* these overdose rescuers obtained naloxone and learned to use it through their social networks, most likely by others trained in the Massachusetts’ programs. Our findings are consistent with and compliment a qualitative study in Baltimore, among OEND participants (N = 25), which found that educating family and other social networks members about overdose and naloxone use was valued by people who use drugs as well as their drug using and non-drug using social network members and was more acceptable than discussing how to reduce drug use behaviors [48]. The diffusion of overdose training and naloxone rescue kits is also consistent with a study of PWID which reported at 3-month follow-up that 40% of trainees had trained someone else [26]. Several studies have shown that OEND training improved knowledge [20,49] and overdose response actions between pre- and post- training overdose events [36,50]. In this study, however, we found no substantive differences in overdose rescue management by those trained by the OEND program directly compared to those trained via social networks. Prospective studies are warranted to determine how social network training and dissemination should be formally incorporated into program design. Furthermore, it is worthwhile to determine the minimum instruction necessary for a person to appropriately administer naloxone while responding to an overdose, specifically to determine whether naloxone for overdose can be safely distributed as an over-the-counter medication [51]. While this study alone is insufficient to claim that over-the-counter access to naloxone is safe and effective, the results support exploration of this possibility.

### Changes in substance use

In the analysis of the subset of participants from whom we collected substance use information more than once, we found no clear evidence that heroin use increased more than decreased among substance using OEND participants. Heroin was the most commonly used substance among participants and a sizeable minority used other substances in the 30 days prior to enrollment. These findings provide reassurance that training active substance users in overdose management and distributing naloxone rescue kits does not lead opioid users to increase their overall opioid use. Our findings are consistent with OEND program evaluations have showed a decrease in heroin injection [24] or overall drug use [26] after OEND. Because our study was completed with program data from participants in a program that was widely disseminated in needle exchanges, drop-in centers and, in particular, substance abuse treatment programs, rather than among a more homogeneous population of study subjects, it is not surprising that participants had substantial flux in their drug use. Nonetheless, we did not find an overall trend toward increased heroin use, and the largest group actually decreased heroin use.

For each of the other substances, almost half or more did not change their use and many participants were not using substances in addition to heroin at all. Although the dataset did not distinguish those participants using prescription medications with or without a prescription, it is possible that participants using prescription medications, including methadone, buprenorphine, other opioids, or barbiturates/benzodiazepines who increased their use, did so because they were engaged in addiction, medical or mental health treatment and receiving prescriptions for these medications.

The increase in benzodiazepine/barbiturate use in the past 30 days was unexpected, because the OEND training specifically addresses polysubstance use, particularly opioid-benzodiazepine combinations, which is an important risk for overdose [52]. Benzodiazepines/barbiturates were used by less than half of participants, yet they were the most commonly used substances other than heroin. If naloxone provided a “safety net” for heroin users, it should increase heroin use, not benzodiazepine/barbiturate use. The percentage of people who reported increased benzodiazepine/barbiturate may be the result of a concurrent increase in benzodiazepine use, as evidenced by an increase in emergency department visits involving benzodiazepines [53]. Another explanation may be that people who enrolled a second time had increased treatment for comorbid mental health disorders which included benzodiazepine/barbiturate prescription [54]. The relationship between illicit opioid use, other sedating medications like benzodiazepines and addiction treatment is complicated by multiple competing risks and benefits. For example, although detoxification can be a first step to treating a substance use disorder and potentially reducing overdose risk, it often includes the use of comfort medications, like benzodiazepines that are sedating and increase overdose risk. Furthermore, the period immediately following discharge from treatment is one of the highest overdose risk times [55], because opioid tolerance is decreased. Thus the role that benzodiazepines play in the cycle of substance use disorder, detoxification, recovery, relapse and overdose is complicated and warrants further study. OEND programs should continue to stress the risks of poly substance use in overdose prevention education.

### Limitations

This study has several limitations. While we report on a larger sample of OEND participants from more diverse settings compared to previous studies, the study was conducted among program participants using program data. Thus, we are not able to compare outcomes to people who did not receive OEND and therefore are unable to measure the impact of receiving OEND, generally. Among those who were re-enrolled by an OEND program site and/or reported an overdose rescue while requesting a naloxone rescue kit refill, the follow-up data were not collected systematically but rather by convenience, which implies that both successful and unsuccessful overdose rescue reports are likely an undercount. The program information we used for our analysis was not collected for research purposes and it included self-reported stigmatized, illegal behaviors. Yet, trained staff collected the data in settings where active substance use by participants was normative and relationships between participants and staff often predated OEND implementation. Neither names nor contact information was collected, so there were substantial confidentiality protections. There may have been some social desirability bias in the self-reporting of substance use because the OEND training included messages about reducing use, particularly polysubstance use. Yet, the strength of these messages was unlikely to be substantially stronger at subsequent visits than at the first visit and the social desirability bias may be smaller in our program data where collecting information was normative as part of providing services compared to research survey data explicitly collected to measure changes in behavior. The program dataset did not distinguish between prescribed and non-prescribed pharmaceutical substances or the clinical indication for prescribed substances. While we were able to confirm that the untrained overdose rescuers (Figure 1) were not trained by OEND staff, we were unable to assess the content of the information that untrained rescuers received through social networks and the extent to which the information was similar or different than that provided by OEND staff. We were unable to account for environmental factors that may have influenced substance use and addiction treatment access that occurred during the study period, such as the implementation of the Massachusetts health care insurance reform law (mandatory universal health insurance coverage) beginning in 2006, expanded access to MDPH supported buprenorphine treatment in 2006–8, restrictions on distribution of 40 mg methadone for pain in 2008, and reformulation of OxyContin in the fall of 2010 [56]. Finally, we sought to highlight important research questions related to OEND, but prospective studies are required to fully respond to these questions.

## Conclusion

Overdose education and naloxone distribution (OEND) in Massachusetts is an overdose prevention intervention that has been widely implemented by nonmedical public health workers among thousands of people who use drugs and have high drug overdose risk. These high risk drug users have witnessed and successfully responded to hundreds of overdoses. We found few differences in behavior between trained and untrained opioid overdose rescuers, which may warrant consideration of over-the-counter status for naloxone rescue kits in future prospective investigations. We found no clear evidence among participants of overall increased heroin use upon receiving comprehensive OEND services. Randomized controlled trials or prospective cohort studies of OEND with systematic and thorough follow-up are the needed next steps in addressing the structure, content and optimal amount of training to accompany naloxone rescue kits and the effect of OEND on participant drug use. Information and naloxone dissemination among social networks also warrants more investigation. Further study is also needed to understand the nuanced relationship between non-opioid sedating medications and opioid overdose. Nonetheless, concern about increased substance use should not impede the study and expansion of OEND programs or policies, legislation or regulations that support them.

## Abbreviations

APHA: American Public Health Association; MDPH: Massachusetts Department of Public Health; OEND: Overdose education and naloxone distribution; PEPFAR: US President’s emergency plan for AIDS relief; PWID: People who inject drugs; QALY: Quality adjusted life year; UNODC: United Nations office on drugs and crime; WHO: World Health Organization.

## Competing interests

The authors declare that they have no competing interests.

## Authors’ contributions

MDS, EQ and AYW developed the original study design. EQ managed the data and she, ZX, HH and AO performed data analysis, all authors contributed to data interpretation. MDS, AYW and ASA wrote the first draft and all authors contributed to editing. All authors read and approved the final manuscript.

## Pre-publication history

The pre-publication history for this paper can be accessed here:

http://www.biomedcentral.com/1471-2458/14/297/prepub
